# Insect Protein Content Analysis in Handcrafted Fitness Bars by NIR Spectroscopy. Gaussian Process Regression and Data Fusion for Performance Enhancement of Miniaturized Cost-Effective Consumer-Grade Sensors

**DOI:** 10.3390/molecules26216390

**Published:** 2021-10-22

**Authors:** Krzysztof B. Beć, Justyna Grabska, Nicole Plewka, Christian W. Huck

**Affiliations:** Institute of Analytical Chemistry and Radiochemistry, University of Innsbruck, Innrain 80/82, 6020 Innsbruck, Austria; Justyna.Grabska@uibk.ac.at (J.G.); nicple@gmx.de (N.P.)

**Keywords:** near-infrared (NIR) spectroscopy, miniaturized sensor, handheld, protein analysis, partial least squares regression (PLSR), Gaussian process regression (GPR), data fusion, insect protein, artisanal food

## Abstract

Future food supply will become increasingly dependent on edible material extracted from insects. The growing popularity of artisanal food products enhanced by insect proteins creates particular needs for establishing effective methods for quality control. This study focuses on developing rapid and efficient on-site quantitative analysis of protein content in handcrafted insect bars by miniaturized near-infrared (NIR) spectrometers. Benchtop (Büchi NIRFlex N-500) and three miniaturized (MicroNIR 1700 ES, Tellspec Enterprise Sensor and SCiO Sensor) in hyphenation to partial least squares regression (PLSR) and Gaussian process regression (GPR) calibration methods and data fusion concept were evaluated via test-set validation in performance of protein content analysis. These NIR spectrometers markedly differ by technical principles, operational characteristics and cost-effectiveness. In the non-destructive analysis of intact bars, the root mean square error of cross prediction (RMSEP) values were 0.611% (benchtop) and 0.545–0.659% (miniaturized) with PLSR, and 0.506% (benchtop) and 0.482–0.580% (miniaturized) with GPR calibration, while the analyzed total protein content was 19.3–23.0%. For milled samples, with PLSR the RMSEP values improved to 0.210% for benchtop spectrometer but remained in the inferior range of 0.525–0.571% for the miniaturized ones. GPR calibration improved the predictive performance of the miniaturized spectrometers, with RMSEP values of 0.230% (MicroNIR 1700 ES), 0.326% (Tellspec) and 0.338% (SCiO). Furthermore, Tellspec and SCiO sensors are consumer-oriented devices, and their combined use for enhanced performance remains a viable economical choice. With GPR calibration and test-set validation performed for fused (Tellspec + SCiO) data, the RMSEP values were improved to 0.517% (in the analysis of intact samples) and 0.295% (for milled samples).

## 1. Introduction

Insect-based food supply is projected to become widely adopted in the near future [1]. In particular, high-quality proteins can be obtained from insect sources, far superior to those of vertebrates and in many ways being better than those derived from plant products [2,3,4]. However, counterfeiting foods to appear richer in protein than advertised has occurred numerous times across the globe [5,6], with the infamous melamine adulteration scandal being the most publicly recognized worldwide [7]. Furthermore, maintaining adequate food quality is one of the key issues identified globally as the goal of contemporary analytical chemistry [8,9,10]. Therefore, developing effective quality control techniques for insect-based food products seems essential to help establishing insect protein as a credible source of healthy food. It lies in keen interest for the public, as in the western world insect products often evoke disgust and rejection [11,12]. A shift in eating habits can lead to a change in the food industry, reduce greenhouse gas emissions and help with improving the environmental footprint [13,14].

Current focus in developing modern analytical methods for foods is oriented at spectroscopic methods [15,16]. Near-infrared (NIR) spectroscopy is a highly potent tool in such role, owing to its fundamental suitability to be used without sample preparation, resulting in its ability to perform rapid, cost-effective, flexible yet reliable analyses in which multiple parameters of foodstuff describing its quality may be determined [17]. NIR spectroscopy is a vibrational spectroscopic technique typically ascribed to the wavelength range between 800 and 2500 nm, corresponding to 12,500–4000 cm^−1^ range on the wavenumber axis of the electromagnetic spectrum, i.e., located between the visible (VIS) and the infrared (IR) regions [18]. The absorption of the radiation in NIR region results from excitations of non-fundamental vibrational modes of molecules, overtones and combinations. This sets NIR spectroscopy apart from the IR technique, which is focused on fundamental modes [19]. Since the molar absorptivity values corresponding to overtones and combinations bands are significantly lower than those of the fundamentals [18], bulk samples may be directly measured. By contrast, IR spectroscopy typically requires diluting the sample in a non-absorbing medium, e.g., KBr powder, to acquire a properly resolved spectrum, unless the attenuated total reflection (ATR) technique is used [20]. The latter limits the sensing of the sample to its immediate surface and requires a contact mode (i.e., sample directly deposited on the ATR crystal), while NIR spectroscopy features deeper radiation penetration depth and delivers the information from a larger volume of the sample in non-contact mode. Moreover, NIR spectroscopy is well-suited to interrogate wet materials [21]. Hence, non-invasive measurements of intact inhomogeneous samples constituting complex matrix, features common for agri-food items, can be performed. This circumstance is accompanied by the beneficial fact that a manifold of structural information is captured as the non-fundamental excitations are significantly more numerous than the fundamental ones and thus NIR signal carries remarkably rich information on the sample [22]. 

Through those values of the physicochemical and practical nature, NIR spectroscopy has become well established in several fields, and in agri-food sector in particular. Rich literature has appeared demonstrating the potential of NIR spectroscopy in analyzing a variety of raw materials and shelf products, e.g., grains [23,24], fruits [25,26], vegetables [27,28,29], rice [30,31], dairy products [32,33,34], meat [35,36], juice [37], or beverages [38,39] among bountiful other studies reported in this field [17].

Food production and supply chain is inherently complex and multi-staged. With accelerating increase in the world food trade and diversification of the supplies, the risk of food quality compromises becomes greatly enhanced. Thus, the development of flexible analytical methods applicable on-site, i.e., at any stage of the supply chain, is prioritized [40]. In this context, the appearance of miniaturized NIR spectrometers in the past decade marked a decisive step up in the potential of this technique [41]. These portable sensors find particular usefulness in the analysis of agri-food items [42]. However, miniaturization imposes use of the technology and engineering solutions that are radically different from those used in benchtop NIR spectrometers. The generic design of a Fourier transform NIR (FT-NIR) spectrometer employs a Michelson or, less often, polarization interferometer in the role of the wavelength selector. By contrast, miniaturized instruments implement diverse wavelength selection principles. While some of these instruments employ the FT-NIR principle, many other solutions have been proved to be competitive in the regime of miniaturization. Popular examples of handheld NIR spectrometers using the Hadamard-transform principle, Fabry–Perot filter, dispersive grating combined with digital micro-mirror device (DMD) can be given for the optical configurations employing single-element detectors [42]. Further, miniaturized NIR instruments designed as multi-channel spectrometers equipped with array detectors are found on the market [42]. Dictated by the limitations of the chosen optical configuration, the size of the spectrometers can range from over 1 kg (and more for certain suitcase-format spectrometers, e.g., portable FT-IR/FT-NIR spectrometers [43]) to pocket-size devices such as NIRONE Sensor S weighing ca. 15 g [42]. New generation of ultra-miniaturized NIR sensors reach less than 1 g of weight [42]. Furthermore, a cost-per-unit of such instruments varies widely from the levels typical for scientific-grade equipment to inexpensive sensors oriented at the consumer market [41,42]. Attention should be given in particular to the latter class, as their potential suitability for consumer use in everyday food quality assessment fits very well the criteria discussed above.

However, the diversity in the employed technology as well as in the unit price leads to a profound instrumental difference manifested among the miniaturized NIR spectrometers, primarily manifested in the operational wavelength regions, spectral resolution and signal-to-noise ratio (SNR). Importantly, this directly translates to marked differences in analytical performance as well as applicability to a particular analytical scenario [41,42]. Therefore, systematic feasibility studies aimed at assessing the limits of applicability and analytical performance, measured by the statistical metrics describing the calibration and prediction accuracy [44,45,46], are in keen interest of the contemporary analytical NIR spectroscopy [41].

The aim of this work is to develop an NIR spectroscopic method for the on-site non-destructive quality control of handmade protein-rich fitness bars in which edible material derived from insects is used. Such products are advertised as modern, healthy and environmentally friendly and are expected to become remarkably popular in the nearest future. Such artisanal food items are suitable to be produced by smaller manufactures and meet the current culinary market trends and are sought by customers [47,48]. However, the quality control of such handcrafted fitness bars with respect to protein content may be seen as economically challenging. Current standard analytical method for protein analysis, the Kjeldahl method, is extremely time and resource inefficient and entirely unsuitable for wider use by smaller manufactures. In this role, NIR spectroscopy offers considerable potential, particularly with the availability of cost-effective miniaturized sensors. Such devices, offered for sub USD 1000 price, are controlled via smartphone and include operating software with pre-calibrated chemometric models that are intended to be operated by untrained personnel [41,42]. As the time-to-result of a single analysis performed by these sensors is measured in seconds, highly affordable quality control could potentially be feasible even for small-scale production lines, provided that satisfactory robustness of the analysis can be maintained by those instruments. To date, no literature studies oriented at the assessment of the analytical performance of miniaturized NIR spectrometers in analyzing insect protein content in oat-based fitness bars have been reported.

The presented investigation included one benchtop FT-NIR (Büchi NIRFlex N-500) and three handheld NIR spectrometers (MicroNIR 1700 ES, Tellspec Enterprise Sensor, and SCiO Sensor) that were systematically evaluated and optimized in their performance in quantitative analysis of protein content in hand-crafted fitness bars based on edible material derived from insects. The considered NIR spectrometers significantly differ by technical principles, operational characteristics and cost-effectiveness. Therefore, evaluating their applicability to this analytical scenario is particularly interesting. Special attention was given to the applicability of consumer-oriented miniaturized NIR spectrometers (Tellspec and SCiO sensors) to this analysis, which could be used by public for the assessment of food products containing proteins derived from insects. These fitness bars feature chocolate coating of uneven thickness across the bar surface. Hence, the samples were analyzed intact and after preparation, to assess whether any differences in the accuracy of quantification appear between the non-destructive and destructive approach. Spectra pretreatments and chemometric algorithms were systematically evaluated towards the best performance for each of the examined sensors. Calibration and external (i.e., test-set) validation were performed using partial least squares regression (PLSR) and Gaussian process regression (GPR) separately for intact and milled samples and for each of the used spectrometers. Finally, the suitability of the data fusion concept was evaluated, in which the spectral data sets obtained by Tellspec and SCiO sensors were combined and used simultaneously in the calibration and prediction. This concept is promising, as the fused spectra cover largely complementary wavenumber regions, and the high cost-effectiveness of Tellspec and SCiO sensors makes their simultaneous use economically feasible.

## 2. Discussion

### 2.1. Sensor Suite

The benchtop instrument used in this study was NIRFlex N-500 (Büchi, Flawil, Switzerland), a FT-NIR spectrometer equipped with a polarization interferometer. Three portable handheld instruments (MicroNIR 1700 ES (Viavi, Milpitas, CA, USA); Enterprise Sensor (Tellspec, Toronto, Canada); SCiO Sensor (Consumer Physics, Tel Aviv, Israel)), each based on a different optical principle, were employed as well. Table 1 summarizes the main technical parameters of these spectrometers.

The benchtop instrument (NIRFlex N-500) offered the widest operational spectral region and the highest resolution. Additionally, as demonstrated in numerous previous studies, it offers very high stability and tends to achieve the highest analytical accuracy in quantitative analyses performed in complex organic matrices characteristic of natural products [42,44,45,46]. Further, the NIRFlex N-500 is equipped with a suite of interchangeable accessories to perform measurements; in this study, the fiber probe accessory (Büchi ‘Fiber Optic Solids’) was employed to perform the surface scan for intact and single-cut bars. The accessory for measuring powder solids (Büchi ‘Solids XL’) in an optical glass cell (cylindrical, Ø 25 mm) was used for the measurement of ground samples. In the latter mode, a sample rotation accessory was used, which is intended to achieve better averaging of the spectra over the volume of the interrogated sample. These features offered the best optimized measuring conditions, maximizing the performance of the analysis performed by the NIRFlex N-500 spectrometer. Given these advantages, the benchtop spectrometer was considered to be the reference NIR instrument, against which the performances delivered by the handheld sensors were evaluated.

MicroNIR 1700 ES is a very compact spectrometer designed with a decisively different optical principle [41,42]. It uses an array detector combined with a linear variable filter (LVF), creating a multi-channel spectrometer in which 128 wavebands are measured simultaneously. Thus, markedly rapid scanning times are offered by this instrument. It constitutes a temperature correction function, which increases its stability of operation over time. Given the very low weight, and thus thermal capacity of this sensor, this function is an essential improvement compared with earlier variants of the spectrometer [49]. Despite a narrower operational spectral region and inferior spectral resolution in relation to those featured by benchtop spectrometers, MicroNIR 1700 ES offers consistently good performance levels in most analyses [42,44,45,46]. While compact, the device still requires constant connection with a host notebook PC to operate, as both the power delivery and control commands and data transfer are handled through the USB interface. However, autonomous, self-powered variants with the essential components shared with this instrument are available as well [50].

The Tellspec Enterprise Sensor is based on InnoSpectra NIR-S-G1 design, which belongs to a different class of instruments oriented at a much wider market with its price per unit being roughly ten times lower than MicroNIR 1700 ES. Thus, it represents the sensors that may be particularly attractive for small manufactures producing artisanal foods. Its operating principle is based on the generic scheme of the conventional dispersive grating spectrometer. However, the wavelength selection is performed using the digital micromirror device (DMD) engineered in micro-scale through micro-electro-mechanical systems (MEMS) technology. The DMD element enables the use of a stationary dispersive grating, resulting in very compact, mechanically robust sensor. It is equipped with a Li-ion battery of 1000 mAh (3.7 V) capacity and is controlled by a dedicated mobile application (iOS and Android variants), while maintaining connection with the host device (i.e., smartphone or tablet) via a Bluetooth low energy (BLE) interface. The measured spectral data is automatically uploaded to a cloud service, from which it can be downloaded for further use.

In some aspects similar, the SCiO sensor is an even more compact and inexpensive ‘pocket size spectrometer’ intended for wide consumer use. These characteristics were accomplished by implementing a simple optical design based on an array of 12 complementary metal–oxide–semiconductor (CMOS) photodiodes coupled with simple optical bandpass filters as the detector, yielding a simple 12-channel spectrometer. Furthermore, a light emitting diode (LED) is used as the radiation source. While entirely avoiding any moving parts and achieving incomparable cost-efficiency, the sensitivity of the chosen detector and the emission properties of LED source dictate the operational spectral region of the sensor, limited to the visible/short-wave NIR (VIS/SW-NIR) region (13,514–9346 cm^−1^). Importantly, the instrument measures only 12 distinct wavebands, which is distinctly inferior to the spectral resolution levels offered by other spectrometers considered here. SCiO is equipped with an inexpensive and compact Li-ion 150 mAh (3.7 V) battery, which is sufficient given the power efficiency of the optical components implemented in the sensor. Operation of the SCiO also requires the spectra first to be automatically deposited in a cloud service, from which these can be then accessed by the logged in user. Its primary role intended by the vendor is food analysis by ordinary consumers, for which role the control software includes a number of pre-calibrated models aimed to predict key properties of foods, e.g., the content of sugar, fat, proteins or moisture, as well as related quality marker indicating freshness or energetic value. The performance of SCiO in the analysis of food items and natural products may vary greatly, depending on the particular sample and analyzed property [41,45]. The specific spot that SCiO occupies in the market, and also the engineering solutions that need to be implemented to achieve its cost-effectiveness, make it particularly interesting to critically evaluate this sensor’s performance in the analytical scenario considered in the present study.

### 2.2. Spectra Pretreatment and Chemometrics

Prior to the analysis, spectral pretreatment procedures were systematically evaluated to develop the best performing approach for each of the spectrometer and sample conditions considered in this study. Universally applied as the first step, the spectra were converted from reflectance R into log 1/R, i.e., the spectral intensity scale linearly dependent on the amount of absorbing matter [51]. Prior to proceeding with the subsequent steps of the analysis, we performed PCA to confirm the uniformity of the processed spectral data sets (Figure S1 in Supplementary Material).

For each used spectrometer, various spectral pretreatments were evaluated and the optimal set of pretreatments resulting in the best prediction performance was selected. The considered pretreatments included Savitzky–Golay (SG) smoothing and differentiation (first and second derivative) with varying number of smoothing points (SP), standard normal variate (SNV), multiplicative scatter correction (MSC) and detrending, as well as the combinations of those procedures. These optimal pretreatments for each analysis are presented in Section 2.4.

PLSR is a frequently used regression method in quantitative analysis by NIR spectroscopy [52,53]. Nonetheless, the predictive performance of PLSR models may be limited in some cases [54]. As demonstrated recently, while PLSR calibration leads to very good results when applied to the spectra datasets obtained with a benchtop instrument, it may offer sub-optimal performance in the case of miniaturized sensors [46]. By contrast, nonlinear quantitative methods, such as GPR, show significant potential for their applicability in more difficult NIR spectroscopic analysis scenarios such as the analysis of complex matrix samples with miniaturized spectrometers [54,55]. Despite that potential, so far relatively little literature data exist on the application of the GPR method to perform analysis of the spectral data acquired by miniaturized NIR sensors. In particular, systematic performance studies taking into account different handheld and laboratory instruments are needed. Therefore, the aim of this work was to conduct a systematic feasibility study that compares the predictive performance of a benchtop and three miniaturized instruments while hyphenated to PLSR and GPR calibration and test-set prediction.

The spectral sets acquired for intact and milled samples were randomly divided into the calibration and validation (i.e., test) sets, with the split ratio of 80:20% in each case, respectively. In the PLSR calibration, a nonlinear iterative partial least squares (NIPALS) algorithm was applied to find the principal components of the analyzed data sets. To minimize the tendency of model overfitting, full (CV) cross validation by means of leave one-out (LOO) framework was performed. The robustness of the calibrated PLSR models was controlled via the root mean square error of cross validation (RMSECV) and validated through external validation by monitoring the root mean square error of cross prediction (RMSEP) value [52].

Gaussian process regression (GPR) calibration and prediction were performed using the rational quartic kernel function of the GPR method, with rational quadratic (RQ) isotropic kernel function and constant basis function. To minimize the overfitting, an out-of-fold CV procedure was applied. Systematic evaluation of the parameters used for CV showed that, above a certain level, negligible improvement is offered by the finer partitioning of the calibration set. The partitioning of each dataset into 15 disjoint subsets was deemed fully adequate for the miniaturized instrument, while 50 subsets were applied in the case of the spectral sets from the benchtop spectrometer. The robustness of the trained model was verified through monitoring RMSECV and RMSEP values.

### 2.3. NIR Spectra of Intact and Prepared Insect Protein Bars

#### 2.3.1. NIR Spectra of Intact Samples

**Figure 1:**
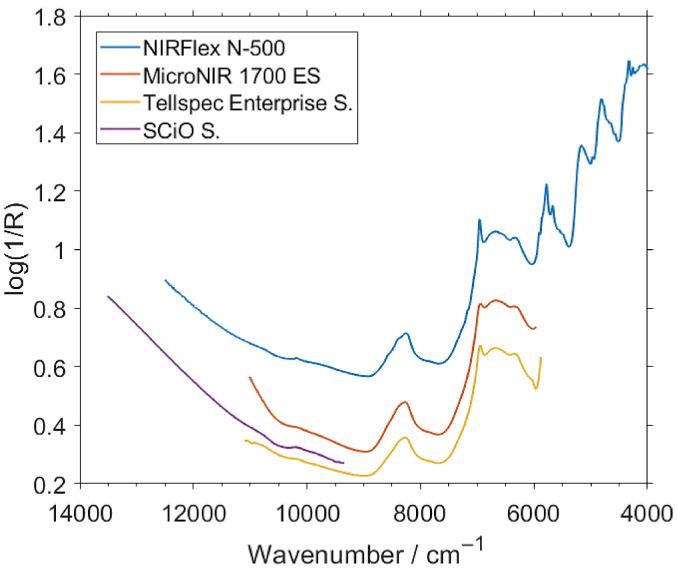
Unpretreated NIR (near-infrared) spectra of exemplary intact samples measured by the spectrometers involved in this study.

The NIR spectra of intact bars were measured in the surface scan mode at six spots at the sample by each spectrometer used in this study. The presence of the chocolate coating of variable thickness and uneven surface introduce understandable difficulties in quantitative analysis, which will be discussed in detail in Section 2.4. Here, a brief look will be made into the features observed in NIR reflectance spectra as well as the differences between the spectral line shapes measured by the benchtop and the three miniaturized spectrometers (Figure 1 and Figures S2 and S3 in Supplementary Materials).

The benchtop spectrometer, Büchi NIRFlex N-500, is able to acquire the entire NIR region (i.e., 12,500–4000 cm^−1^; 800–2500 nm) with the highest resolution, and hence, may be considered to be the reference when dissecting the fine differences by which the spectra obtained by the other sensors are distinguished. The wavenumber region dominated by binary combination bands, between ca. 6000–4000 cm^−1^, can only be acquired by the benchtop spectrometer in the present case. That region of NIR spectrum shows the strongest spectral intensity. However, meaningful absorption bands can be also observed in the 9000–6000 cm^−1^ region of these spectra, which can be acquired by two of the used miniaturized spectrometers as well (Figure 1).

The MicroNIR 1700 ES and Tellspec Enterprise Sensor, which operate in similar spectral regions and with comparable resolutions, demonstrate a large similarity between the measured spectra of the protein bars as well. When compared with the spectra acquired by the benchtop spectrometer, the significantly lower spectral resolution of these two instruments seems not to be noticeable, given the broadness of the absorption features manifested in the examined spectra. The sole easily distinguishable distinction between these spectra appears at ca. 6960 cm^−1^, where the presence of a sharp peak can be properly acquired by the benchtop instrument. The lower resolution of the handheld spectrometers does not allow them to precisely capture the shape of that peak.

The SCiO Sensor operates at a noticeable higher wavenumber spectral region (i.e., VIS/SW-NIR) than the other two handheld instruments, and to a lesser extent, also the benchtop one. However, the N-500 spectrometer provides gradually decreasing S/N levels of the spectra above ca. 10,000 cm^−1^, while rather consistent S/N level in the spectra measured by SCiO across its operational spectral region can be noticed. Furthermore, the fragment of the spectrum of the protein bars observed by SCiO device is relatively flat with absence of any well-resolved peaks. Therefore, the significance of the spectral resolution offered by this instrument (undisclosed by the manufacturer) is less critical than it is for the spectrometers focused on the lower wavenumber parts of the NIR spectrum. Thus, it will be particularly interesting to evaluate the performance of SCiO Sensor against the remaining instruments in this analysis.

The general appearance of the NIR spectral line shape of the protein bars resembles well the typical spectrum of oats or grains [56,57,58]. The chemical similarity of the matrix in this case is anticipated, as oat is one of the major substrates used in the production of the bars. Understandably, common organic matter present in foodstuff, i.e., carbohydrates, fats and moisture, among others should be anticipated to contribute to the measured NIR spectra as well. Following the literature, the absorption features up to the third overtones observed in the spectra can be roughly assigned to these common constituents [59]. Albeit, to an extent depending on the matrix, the signal corresponding to the protein content may be expected to be located in the following wavenumber regions: 8500–8400–8100, 6700–6600, 5950–5800, 5250–5100, 4800–4650, around 4500, 4400–4200 and around 4050 cm^−1^. Carbohydrates mostly contribute to the spectral intensity at 9000–8000, 7100–6000, 5250–5150, 4800–4600, around 4500 and 4300–4200 cm^−1^. The signal from lipids is manifested in NIR spectra at: 9000–8000, 7400–6800, around 6200, 5900–5700, 5250–5200, 4900–4400 and 4350–4000 cm^−1^ [59]. Less apparent is the spectral signature of water present in the samples, as their moisture levels is relatively low; however, one should expect that the typical absorption regions of water, with prominent bands at 7000–6950 and 5200–5100 cm^−1^ [59,60], contribute to the strong spectral intensity of the present spectra (Figure 1). As expected, the complex organic matrix yields highly convoluted NIR spectrum, in which the absorption bands of the major constituents overlap. Typically, it is not a critical issue, as MVA calibration can successfully elucidate the spectral information correlated just with the quantified contents. Towards the upper part of the spectra, i.e., above ca. 9000 cm^−1^, the scattering effects manifested by the elevation of the baseline can be observed, which likely result from the granularity of the interrogated sample. This effect is easily corrected by the spectra pretreatments.

#### 2.3.2. NIR Spectra of Milled Samples

**Figure 2:**
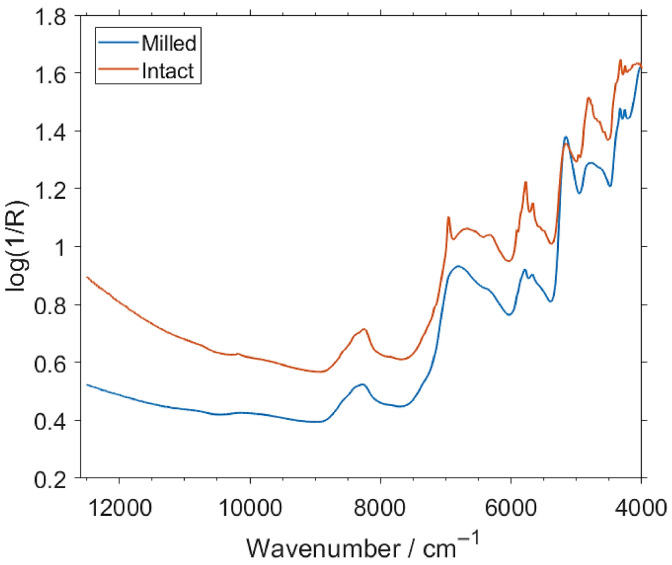
Exemplary spectra (prior any pretreatments) of intact and milled insect protein bars measured by the benchtop spectrometer Büchi NIRFlex N-500.

The NIR spectra of milled samples retain in majority the features observed in the spectra of intact bars (Figure 2). For clarity, the differences in the spectra of the protein bars resulting from milling will be discussed on the example of the measurements performed by the benchtop spectrometer, as it offers the widest spectral region and the highest resolution (Table 1). As shown in Figure 2, the change in the sample state and the measurement conditions lead to noticeable differences in the respective NIR spectra.

The visible differences in the spectral line shape should be primarily associated with the spectral footprint of the chocolate coating that strongly contributes to the spectra measured for intact bars (Figures S2 and S3 in Supplementary Materials). The most apparent features related to the coating can be observed at ca. 6960–6300 cm^−1^, where the shape of the observed broad absorption band and an outstanding peak appears in the spectra of intact samples. In this region, the strong presence of the first overtone of OH stretching vibration is known [61]. A slightly less pronounced change in the spectral line shape is visible at ca. 4900–4800 cm^−1^, where a distinct peak at 4824 cm^−1^ appears in the spectra of intact samples. In this region, the manifestation of the strong combination bands involving OH stretching vibration is expected [61], also known to be clearly visible in the NIR spectra of sugars and sucrose in particular [37,62]. Hence, the observed difference may be associated with the high sugar content present in the chocolate coating, as anticipated.

### 2.4. Comparison of the Analytical Performance of the Benchtop and Miniaturized NIR Spectrometers in the Prediction of the Total Protein Content in Insect Protein Bars

#### 2.4.1. Intact Samples

Table 2 presents the results for the optimized analytical procedure for the prediction of the total protein content in intact protein bars by the considered benchtop and miniaturized NIR spectrometers. The resulting performance unveils that the rapid non-destructive analysis is feasible, with RMSEP values not exceeding 0.659% for the PLSR analyzed protein concentration range. However, it may be noticed that the model fit quality reflects a relatively high random sample-to-sample variance, resulting from the presence of chocolate coating of strongly varying thickness across the bar’s surface.

The performance of the calibration and prediction was noticeably improved with the use of the GPR method, with the exception of the SCiO sensor. For the remaining instruments, the improvement of roughly 21%, 29% and 14% for, respectively, N-500, MicroNIR 1700 ES and Tellspec Enterprise Sensor was noted with GPR calibration. 

The similarity of the RMSEP values among all evaluated spectrometers as well as the level of improvement between the PLSR and GPR calibrations also suggest that the primary limiting factor was related to the sample property and less attributed to the instrumental differences. Furthermore, the spectral sets required rather considerable extent of pretreating. In particular, relatively strong smoothing had a clearly positive effect on the performance, with the exception of MicroNIR 1700 ES, which did not suffer much from this scanning mode.

#### 2.4.2. Milled Samples

Successful analysis of the total protein content in chocolate-coated insect protein bars was deemed feasible through the coating in the non-destructive way. However, the observed features of the calibration models suggested that such sample presentation is a limiting factor for the potential optimal performance of each of the considered NIR spectrometers. Therefore, milled samples were analyzed as well, with the resulting performance summarized in Table 3. Noteworthy, the best performance as evidenced by RMSEP values decreased decisively in each case for the milled samples. Furthermore, the quality of the measured spectra improved markedly, and the spectral sets required relatively less excessive pretreatments. With the intrinsic properties of the samples becoming relatively less meaningful for the optimal performance of each sensor, this analysis provides better ground for the assessment of the instrumental difference as well as the MVA calibration method.

With the PLSR calibration, the benchtop spectrometer (NIRFlex N-500) performed better in analyzing milled samples compared with intact ones; the RMSEP improved to 0.210% (from 0.611% for intact samples), i.e., 2.9 times. This demonstrated the general improvement achievable in NIR spectral analysis when eliminated are the limitations that resulted from the randomness of the chocolate coating and possible internal inhomogeneity in the bars. However, the PLSR calibration did not improve as much for the miniaturized sensors, with the best performing handheld instrument (MicroNIR 1700 ES) achieving RMSEP of 0.525% vs. 0.620% for the intact samples. 

**Figure 3:**
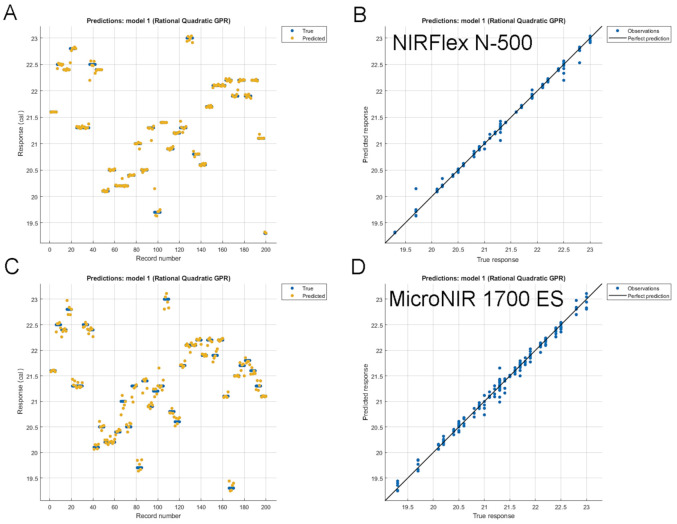
The fit of the GPR model to the calibration set of milled samples for (**A**,**B**) benchtop and (**C**,**D**) miniaturized MicroNIR 1700 ES spectrometer. (**A**,**C**): response plot; (**B**,**D**): predicted vs. true response plot.

In the previous study [46], we demonstrated that PLSR calibration did not allow to maximize the performance of the miniaturized spectrometers, while more advanced non-linear MVA methods, such as GPR or artificial neural networks (ANN), demonstrated higher potential to elucidate the correlated information from the spectral sets acquired by miniaturized instruments. In particular, GPR calibration showed promising results, reconciling very high accuracy and straightforward applicability. In the present case, the analysis of milled samples with GPR has resulted in the noticeable improvement of the prediction performance of the handheld instruments, with the fit quality of the model to the calibration set comparable to that of the benchtop spectrometer (the example of MicroNIR 1700 ES is given in Figure 3). MicroNIR 1700 ES also offered the best prediction performance with the lowest RMSEP, surpassing that of the benchtop spectrometer. In that case, the improvement in RMSEP to 0.230% from 0.525% (i.e., ca. 2.3 times) was demonstrated. Tellspec Enterprise Sensor and SCiO Sensor also yielded decisively higher accuracy as manifested by, respectively, RMSEP values of 0.326% and 0.338%, i.e., 1.75 and 1.68 times lower than those offered by PLSR. 

Thus, in the present analytical scenario, hyphenation of miniaturized NIR spectrometers with GPR calibration yields measurable gains in their analytical performance.

### 2.5. Performance Enhancement of the Cost-Effective Miniaturized NIR Spectrometers by Data Fusion

As discussed in the Introduction Section, the analytical application explored in this study would potentially benefit from the maximized applicability of cost-effective NIR miniaturized spectrometers oriented at the consumer market, such as Tellspec and SCiO sensors considered here. While the predictive performance of these two instruments was fully satisfactory in every case presented here, when the instrument-independent conditions for the analysis were assured (that is, the sample preparation and optimal MVA calibration method, i.e., analysis of milled samples using GPR), these two sensors offered the performance levels evidently inferior not only to the benchtop NIRFlex N-500 but also to the miniaturized MicroNIR 1700 ES instruments (Section 2.4.2 and Table 3). Hence, it is likely that the performance limits governed by the instrumental difference were exposed in that case. This is entirely understandable, considering the vastly disparate markets and affordability levels of the compared spectrometers. However, this also shows the potential for further improvements.

**Figure 4:**
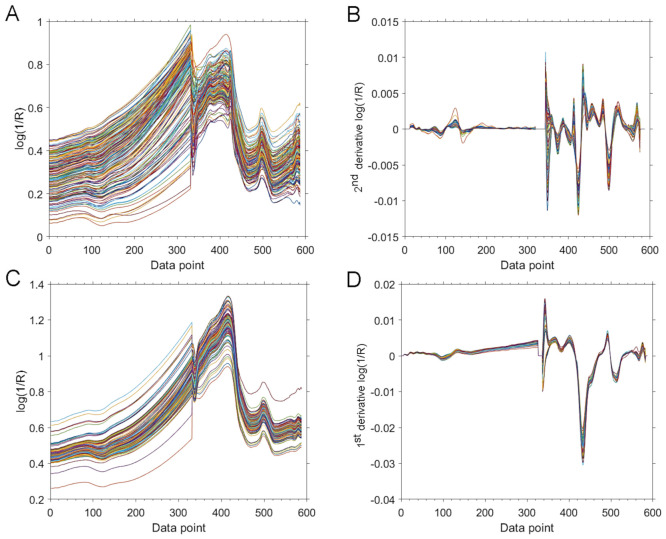
The fused spectra from the two cost-effective miniaturized NIR spectrometers (Tellspec Enterprise Sensor and SCiO Sensor) for intact (**A**,**B**) and milled (**C**,**D**) samples. A and C: unpretreated fused spectra. (**B**,**D**): fused spectra after pretreatments (SNV + SG2 with 25 SP and SG1 with 11 SP).

Tellspec and SCiO sensors operate in largely complementary spectral regions, with only moderate overlap between 11,111–9346 cm^−1^ (Figure 1). Combined, these two sensors cover considerably wider spectral region of 13,514–5888 cm^−1^. Therefore, a calibration and prediction procedures performed for a fused spectral data set provided by these two sensors is a promising concept worth evaluating towards the prediction performance. In this case, the spectral sets fused from the Tellspec and SCiO sensors (Figure 4A,C) were analyzed with the use of the pretreatments determined as optimal for both sensors, separately for the case of the intact (Figure 4B) and milled samples (Figure 4D). The fused spectral sets were subsequently used for PLSR and GPR calibration and test-set validation (Table 4).

As demonstrated, the prediction performance offered by GPR applied to the fused data (Tellspec + SCiO) is measurably improved for both the intact and the milled sample analysis. In the case of the former, the RMSEP value was decreased to 0.517% (Table 4) from the values of 0.578% and 0.580% achieved by each of these sensors separately (Table 3). In the case of the milled samples, the RMSEP for the GPR prediction from the fused spectral set was improved to 0.295% (Table 4) compared with 0.326% and 0.338% offered by these instruments separately (Table 3). In both cases the ratio of the performance enhancement was above 10%. Thus, future considerations of applying the data fusion concept when using more than one miniaturized NIR spectrometers seem viable further systematic developments towards the performance of the analysis. It should be noted that the price per unit of these two sensors combined is still decisively lower than that of e.g., MicroNIR 1700 ES.

With PLSR calibration, the fused spectral sets (Tellspec + SCiO) did not offer an improvement in prediction of the test-set, with RMSEP values remaining slightly inferior to the analysis performed for these two sensors independently (Table 4). Therefore, it seems that the potential of the GPR method to tackle with more challenging spectral data sets, as evidenced in the previous study [46], makes it suitable to better elucidate the intended information from fused spectra as well. 

## 3. Materials and Methods

The fitness bars based on insect protein were purchased from the producer located in Germany. The bars are hand-crafted and contain oat flakes, insect and pea protein, nuts, and honey, and are coated in Belgian chocolate. They are available in four flavors: peanut-cranberry (EC), hazelnut-cocoa (HK), macadamia-salted caramel (MS) and cashew-blueberry (CB), as well as in the limited winter edition ‘Omas Apfelstrudel’ (OA). In total 40 bars, 8 of each flavor, were acquired for the purpose of this study. Depending on the variety, the bars nominally contain 23.5–24.0% (*w*/*w*) protein with 12% being the protein derived from buffalo worm. According to the manufacturer, the buffalo worms used for the protein production are grown under their natural living conditions without the use of pesticides, hormones, antibiotics or preservatives.

The spectra of each of the 40 samples were recorded with the four NIR spectrometers (Section 2.1). The measurements of intact bars were performed in diffuse reflectance mode at 6 different locations on the bar’s surface; 3 measuring points were on the top of the bar, followed by 3 measuring points on the bottom, which resulted in a total of 6 spectra acquired per sample. The chocolate coating of the bars varied in thickness of the layer and in the amount of the covered area. Therefore, a method of spectra acquisition involving sample preparation (grinding) was evaluated as well to enable measuring the spectra of the bars without the influence of the chocolate coating of uneven thickness. The grinding of the samples to high fineness was performed using Retsch ZM 200 (Retsch, Haan, Germany) centrifugal mill, at 10,000 rpm with additional cooling to prevent overheating of the ground material. For the ground samples, the measurements were performed in in an optical glass cell (Hellma GmbH & Co. KG, Müllheim, Germany) of 25 mm diameter. Automatic sample rotation accessory was used with NIRFlex N-500 while for the miniaturized spectrometers, the cell was manually rotated between the measurements; 6 spectra per sample on each spectrometer were acquired. Effectively, 240 spectra were acquired in each case.

The spectral measurements on the benchtop spectrometer NIRFlex N-500 were performed in 12,500–4000 cm^−1^ region, with 32 scans collected per each spectrum at 8 cm^−1^ spectral resolution interpolated to 2126 data points. Intact bars were measured in surface scan mode using a fiber probe accessory—‘Fiber Optic Solids’ (Büchi, Flawil, Switzerland) while ‘Solids XL’ accessory with sample rotation accessory was used for milled samples placed in the cell. 

The measurements with MicroNIR 1700 ES were performed with 7.5 ms integration time and 1000 scans accumulated per each spectrum; each spectrum constituted 125 spectral points between 11,013–5967 cm^−1^, with average data spacing of 37 cm^−1^. During the measurements, the temperature correction function implemented in the instrument was active.

The control software for both Tellspec Enterprise Sensor and SCiO Sensor offer no possibility to adjust any of the operational parameters of the instruments; thus, the factory default options were applied. Tellspec Enterprise Sensor presents to the user the spectra in 256 data points, covering 11,111–5882 cm^−1^ region with an average data spacing of 13 cm^−1^. In the case of SCiO Sensor, the spectra presented to the user consist of 331 spectral points, spanned over 13,514–9346 cm^−1^ range.

For the purpose of the calibration of the quantitative MVA models, reference analysis of the total protein content present in the samples was performed by means of the Kjeldahl method [63].

Spectral pretreatments and PLSR calibration and prediction were carried out using “The Unscrambler X Version 10.5”. The calibrations and predictions using Gaussian process regression (GPR) were performed in MATLAB R2018b environment (The MathWorks Inc., Natrick, MA, USA), using the Statistics and Machine Learning Toolbox.

The final performance and the robustness of all developed calibration models (PLSR and GPR) was evaluated through the test set validation (TSV) procedure applied to an independent set. The split of the spectra into calibration and TSV sets was conducted in a 200:40 ratio.

## 4. Summary

Benchtop (Büchi NIRFlex N-500) and three miniaturized (MicroNIR 1700 ES, Tellspec Enterprise Sensor and SCiO Sensor), in hyphenation to PLSR and GPR calibration methods, were evaluated and optimized towards their applicability to perform protein content analysis in insect protein fitness bars. NIR spectroscopy, both with the use of benchtop and miniaturized handheld instruments, can be successfully applied for performing rapid, non-destructive analysis of the total protein content. When accepting the destructive way of analysis, further gains in analytical accuracy can be gained. When hyphenated to the PLSR method, the handheld instruments showed a clear inferiority in the prediction performance, evaluated through the RMSEP values determine for independent test-set data. The application of the non-linear GPR method for the calibration improves decisively the accuracy of the miniaturized spectrometers, with MicroNIR performing on par with the benchtop instrument, and Tellspec and SCiO sensors being only moderately inferior.

While the analyzed protein content was between 19.3–23.0% in the calibration set, the RMSEP values for the non-destructive analysis of the intact bars were 0.611% (benchtop) and remained in the range of 0.545–0.659% (miniaturized) for PLSR, and 0.506% (benchtop) and 0.482–0.580% (miniaturized) for GPR analysis. When considering milled samples, the respective RMSEP values for PLSR prediction improved to 0.210% for benchtop spectrometer but remained in the inferior range of 0.525–0.571% for the miniaturized ones. The RMSEP values for GPR prediction based on the miniaturized spectrometers were improved to 0.230% (MicroNIR 1700 ES), 0.326% (Tellspec) and 0.338% (SCiO). Considering that Tellspec and SCiO sensors cover largely complementary regions of VIS/SW-NIR and NIR wavelengths, an attempt to perform data fusion from these sensors was made. With the GPR calibration and test-set validation performed for fused (Tellspec + SCiO) data, the RMSEP values were improved to 0.517% (in the analysis of intact samples) and 0.295% (for milled samples). Considering that the Tellspec and SCiO sensors are consumer-oriented devices, their combined use for enhanced performance remains a viable economical choice.

## Figures and Tables

**Table 1:** Technical parameters of the spectrometers used in this study.

Spectrometer (Vendor)		Key Components		Spectral Region		Resolution [nm]	Connectivity (Data Transfer)	Dimensions [cm]	Weight [g]
	Source	Wavelength Selector	Detector	[nm]	[cm^−1^]				
**NIRFlex N-500** **(Büchi)**	Tungsten halogen (duplicated)	Polarization interferometer (TiO_2_ wedges)	InGaAs (single-element, thermoelectric cooling)	800–2500	12,500–4000	~2	Ethernet	45 × 35 × 25	ca. 35,000
**MicroNIR** **1700 ES** **(VIAVI)**	Tungsten halogen (duplicated)	LVF	InGaAs (array; 128 elements)	908–1676	11,013–5967	12.5	USB—control and power delivery	5.0 × 4.6 (Ø)	58
**Enterprise** **Scanner** **NIR-S-G1** **(Tellspec)**	Tungsten halogen (duplicated)	Stationary dispersive grating and MEMS DMD	InGaAs (single-element)	900–1700	11,111–5882	10	Bluetooth (Cloud service)	8.2 × 6.3 × 4.0	136
**SCiO** **(Consumer** **Physics)**	LED	Bandpass filter	Si photodiode (CMOS) array (12 elements)	740–1070	13,514–9346	Not disclosed	Bluetooth (Cloud service)	6.8 × 3.9 × 1.5	35

Abbreviations: CMOS—Complementary Metal–Oxide–Semiconductor; DMD—Digital Micromirror Device; InGaAs—Indium Galium Arsenide; LED—Light Emitting Diode; LVF—Linear Variable Filter; MEMS—Micro-Electro-Mechanical System; USB—Universal Serial Bus; Si—Silicon, NIR—near-infrared.

**Table 2:** The parameters of the best performing regression models for the analysis of protein content (range: 19.3–23.0% (*w*/*w*)) in intact bars.

PLSR				
	Benchtop	Miniaturized		
	NIRFlex N-500	MicroNIR 1700 ES	Tellspec Enterprise Sensor	SCiO Sensor
Pretreatment	SG2 (29 SP)	SNV, SG2 (3 SP)	SNV, SG2 (25 SP)	SNV, SG2 (25 SP)
*R*^2^ (Cal)	0.43	0.57	0.38	0.55
*R*^2^ (CV)	0.35	0.47	0.30	0.38
RMSEC [%]	0.641	0.557	0.668	0.568
RMSECV [%]	0.687	0.624	0.716	0.671
*R*^2^ (TSV)	0.49	0.46	0.40	0.59
**RMSEP [%]**	**0.611**	**0.620**	**0.659**	**0.545**
**GPR**				
	**Benchtop**	**Miniaturized**		
	**NIRFlex N-500**	**MicroNIR 1700 ES**	**Tellspec Enterprise Sensor**	**SCiO Sensor**
Pretreatment	SG2 (29 SP)	SNV, SG2 (3 SP)	SNV, SG2 (25 SP)	SNV, SG2 (25 SP)
*R*^2^ (Cal)	0.99	1.00	0.54	1.00
*R*^2^ (CV)	0.42	0.53	0.33	0.52
RMSEC [%]	0.083	0.00015	0.579	0.00014
RMSECV [%]	0.65	0.59	0.70	0.60
*R*^2^ (TSV)	0.65	0.68	0.56	0.54
**RMSEP [%]**	**0.506**	**0.482**	**0.578**	**0.580**

SG—Savitzky–Golay (1, 2—first, second derivative); SP—Smoothing Point; SNV—Standard Normal Variate, PLSR—partial least squares regression, RMSEP— the root mean square error of cross prediction, GPR—Gaussian process regression.

**Table 3:** The parameters of the best performing regression models for the analysis of protein content (range: 19.3–23.0% (*w*/*w*)) in milled bars.

PLSR				
	Benchtop	Miniaturized		
	NIRFlex N-500	MicroNIR 1700 ES	Tellspec Enterprise Sensor	SCiO Sensor
Pretreatment	SG (5 SP)	SG1 (7 SP)	SG1 (11 SP)	SG1 (11 SP)
*R*^2^ (Cal)	0.96	0.89	0.65	0.54
*R*^2^ (CV)	0.88	0.80	0.52	0.46
RMSEC [%]	0.182	0.286	0.505	0.581
RMSECV [%]	0.309	0.382	0.591	0.630
*R*^2^ (TSV)	0.94	0.62	0.55	0.55
**RMSEP [%]**	**0.210**	**0.525**	**0.571**	**0.568**
**GPR**				
	**Benchtop**	**Miniaturized**		
	**NIRFlex N-500**	**MicroNIR 1700 ES**	**Tellspec Enterprise Sensor**	**SCiO Sensor**
Pretreatment	SG (5 SP)	SG1 (7 SP)	SG1 (11 SP)	SG1 (11 SP)
*R*^2^ (Cal)	1	0.99	0.99	0.99
*R*^2^ (CV)	0.87	0.99	0.84	0.93
RMSEC [%]	0.0011	0.0006	0.0002	0.0003
RMSECV [%]	0.3150	0.0782	0.3397	0.2248
*R*^2^ (TSV)	0.91	0.94	0.87	0.84
**RMSEP [%]**	**0.266**	**0.230**	**0.326**	**0.338**

SG—Savitzky–Golay (1, 2—first, second derivative); SP—Smoothing Point, PLSR—partial least squares regression, RMSEP— the root mean square error of cross prediction, GPR—Gaussian process regression.

**Table 4:** Regression models constructed for the fused spectra from the miniaturized NIR spectrometers (Tellspec Enterprise Sensor and SCiO Sensor) for the analysis of protein content (range: 19.3–23.0% (*w/w*)) in intact and milled bars.

	Intact		Milled	
	PLSR	GPR	PLSR	GPR
Pretreatment	SNV, SG2 (25 SP)	SNV, SG2 (25 SP)	SG1 (11 SP)	SG1 (11 SP)
*R*^2^ (Cal)	0.41	0.9	0.53	0.99
*R*^2^ (CV)	0.28	0.55	0.48	0.9
RMSEC [%]	0.654	0.272	0.580	0.0002
RMSECV [%]	0.723	0.574	0.620	0.263
*R*^2^ (TSV)	0.38	0.64	0.51	0.89
**RMSEP [%]**	**0.671**	**0.517**	**0.596**	**0.295**

SG—Savitzky–Golay (1, 2—first, second derivative); SP—Smoothing Point; SNV—Standard Normal Variate, PLSR—partial least squares regression, RMSEP— the root mean square error of cross prediction, GPR—Gaussian process regression.

## Data Availability

Not applicable.

## Supplementary Materials

The following are available online, Figure S1: (Left) PCA score plot for PC1 vs. PC2 and (Right) the explained variance for the spectral set of milled bars (spectrometer: Büchi NIRFlex N-500). Figure S2: exemplary spectra (prior any pretreatments) of intact and milled bars as well as isolated chocolate coating (spectrometer: Büchi NIRFlex N-500). Figure S3: the exemplary spectra of an intact bar and chocolate coating after baseline offset correction (spectrometer: Büchi NIRFlex N-500).
