# Supplementary material for: Insect Protein Content Analysis in Handcrafted Fitness Bars by NIR Spectroscopy. Gaussian Process Regression and Data Fusion for Performance Enhancement of Miniaturized Cost-Effective Consumer-Grade Sensors
Source: Molecules. 2021 Oct 22;26(21):6390. doi: 10.3390/molecules26216390 (PMC8587585; doi:10.3390/molecules26216390)
Supplement: Supplementary file 1 [file molecules-26-06390-s001.zip › molecules-1352138-supplementary.pdf]

molecules

MDPI

Supporting Information

Insect protein content analysis in handcrafted fitness bars by NIR spectroscopy. Gaussian process regression and data fusion for performance enhancement of miniaturized cost-effective consumer-grade sensors

SUPPORTING INFORMATION

Krzysztof B. Bec[1]*, Justyna Grabska[1], Nicole Plewka[1] and Christian W. Huck[1]*

[1] Institute of Analytical Chemistry and Radiochemistry, University of Innsbruck, Innrain 80/82, 6020 Innsbruck, Austria; (K.B.B.) Krzysztof.Bec@uibk.ac.at; (J.G.) Justyna.Grabska@uibk.ac.at; (C.W.H.) Christian.W.Huck@uibk.ac.at

* Correspondence: Krzysztof.Bec@uibk.ac.at; Christian.W.Huck@uibk.ac.at

Figures (S1 to S3)

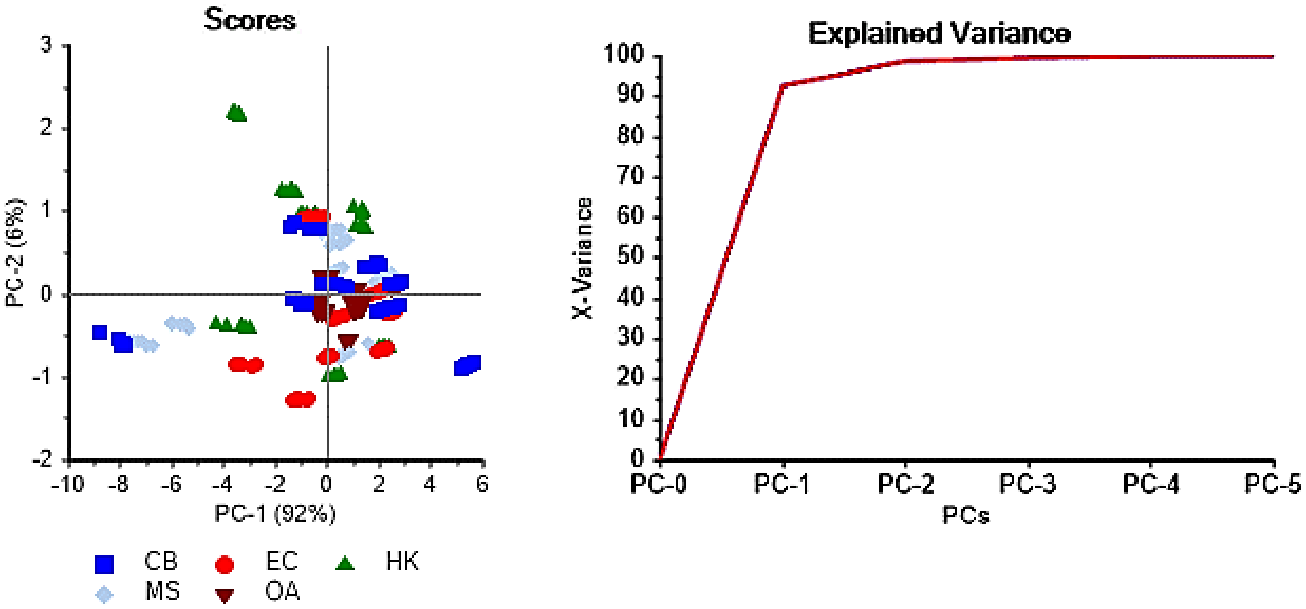

Figure S1. (Left) PCA score plot for PC1 vs. PC2 and (Right) the explained variance for the spectral set of milled bars (spectrometer: Büchi NIRFlex N-500).

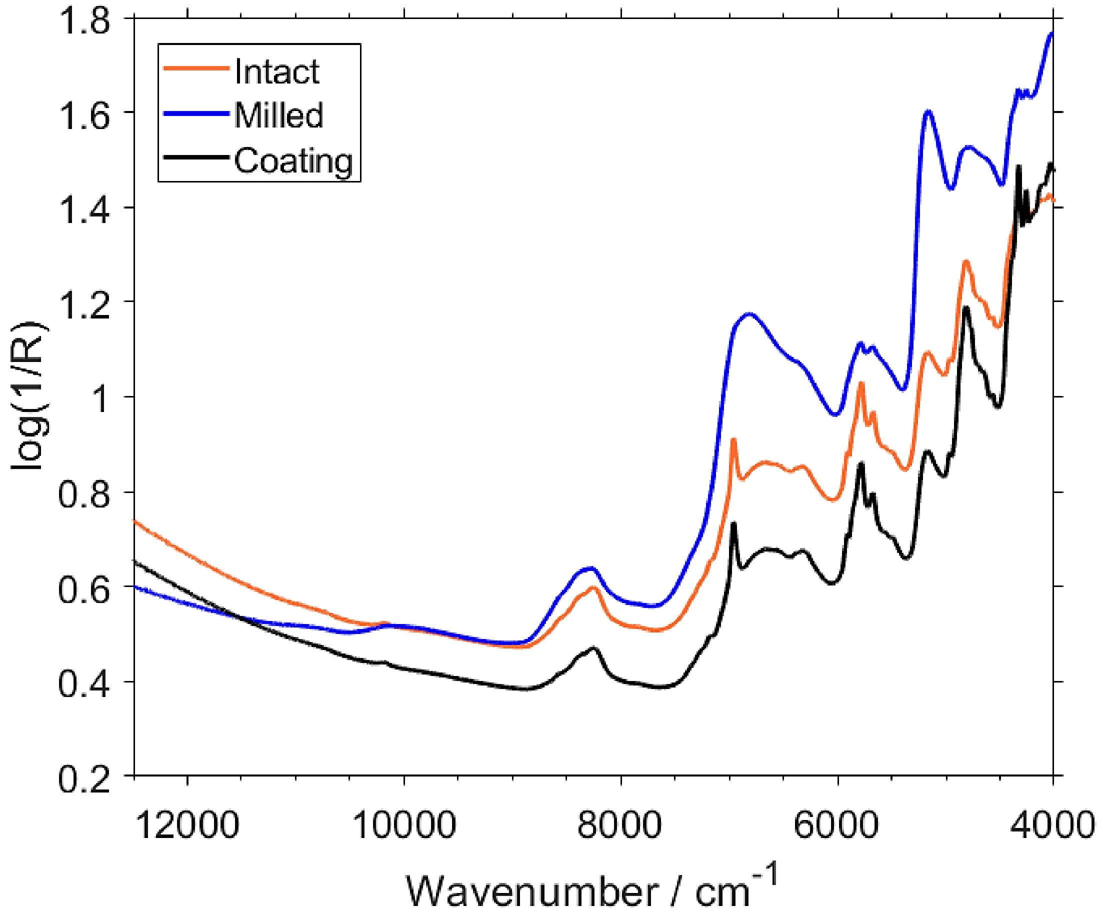

Figure S2. Exemplary spectra (prior any pretreatments) of intact and milled bars as well as isolated chocolate coating (spectrometer: Büchi NIRFlex N-500).

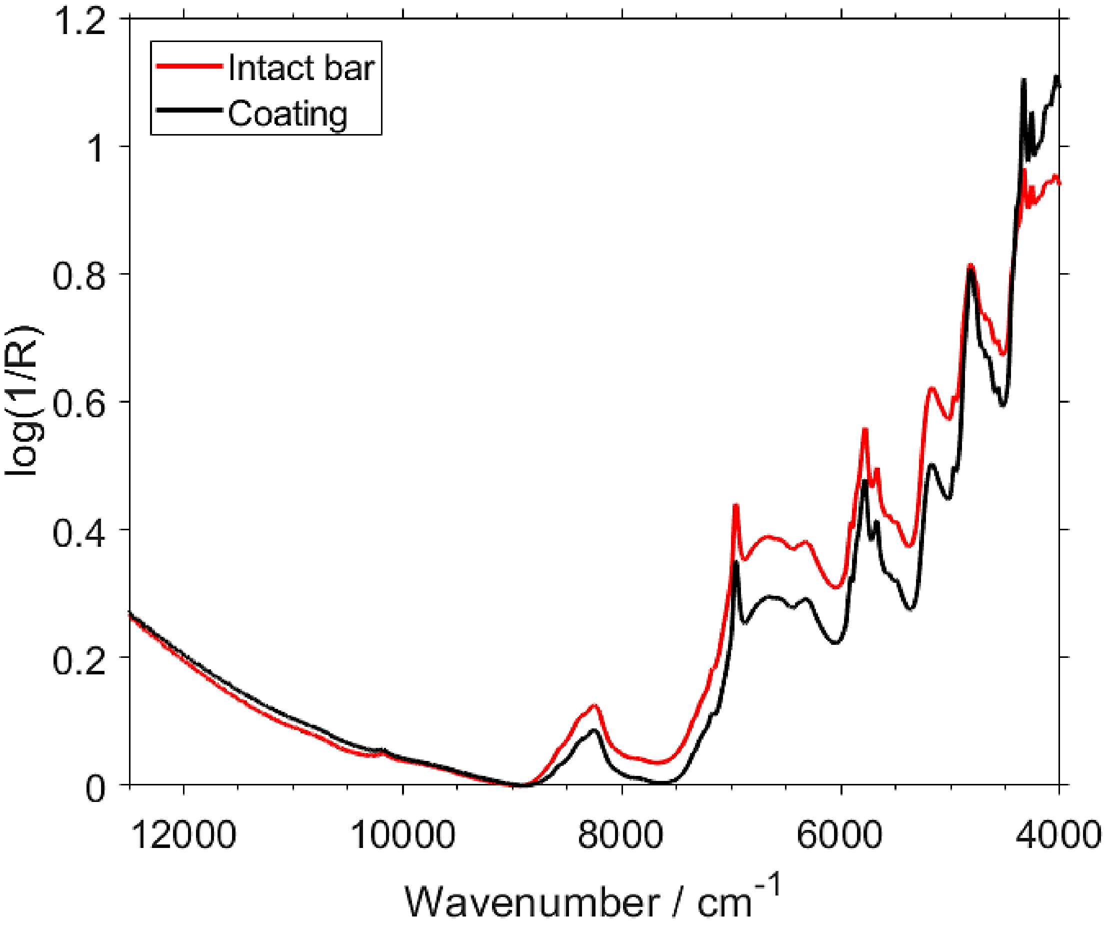

Figure S3. The exemplary spectra of an intact bar and chocolate coating after baseline offset correction (spectrometer: Büchi NIRFlex N-500).
